# Supplementary material for: Discovering new photovoltaics using optimal transport theory
Source: Faraday Discuss. 2026 Jul 21;268:449–63. doi: 10.1039/d6fd00016a (PMC13719145; doi:10.1039/d6fd00016a)
Supplement: Supplementary file 1 [file FD-268-D6FD00016A-s001.pdf]

Supplementary Information (SI) for Faraday Discussions.
This journal is © The Royal Society of Chemistry 2026

Supplementary Information for: Discovering new photovoltaics using optimal transport theory

Matthew A. H. Walker[a], Zibo Zhou[c], Junayd Ul Islam[d], and Keith T. Butler[b,*]

DOI: 10.1039/x0xx00000x

Full details of parameter combinations

For a complete grid search, we considered 21 α values from 0 to 1 in intervals of 0.05, with all combinations of the following method choices.

Structure matrix C:

- Adjacency matrix
- Distance-weighted adjacency matrix
- Shortest path (through distance-weighted adjacency matrix)
- Harmonic distance
- Distance-weighted harmonic distance
- Raw interatomic distances

Where 'distance-weighted' involves taking the adjacency matrix used to derive the other matrices and multiplying it by the matrix of interatomic distances. This provides a compromise between the adjacency matrix, which is simple for the optimiser to handle but is too information-poor, and the raw interatomic distances, which are very informative but too granular, making convergence difficult. We also tried scaling the matrices by dividing them by the mean of their elements to balance the magnitude of the structural and feature-space costs. This improved performance in several cases.

Embeddings:

- One-hot vectors
- Randomly generated distributed vectors
- Magpie[1]
- Oliynik[2]
- SkipAtom[3]
- CrystaLLM[4]

Feature distances d:

- Euclidean distance
- Manhattan (Cityblock) distance

- Cosine distance

Full training data for all combinations is provided in the repository at `https://github.com/matthewww98/FGW_for_PVs`.

MACE embedding construction

In Figure 1 we see that mean pooling of atomic embeddings for a graph-level embedding leads to better performance than sum pooling, as we might expect from an intensive property.

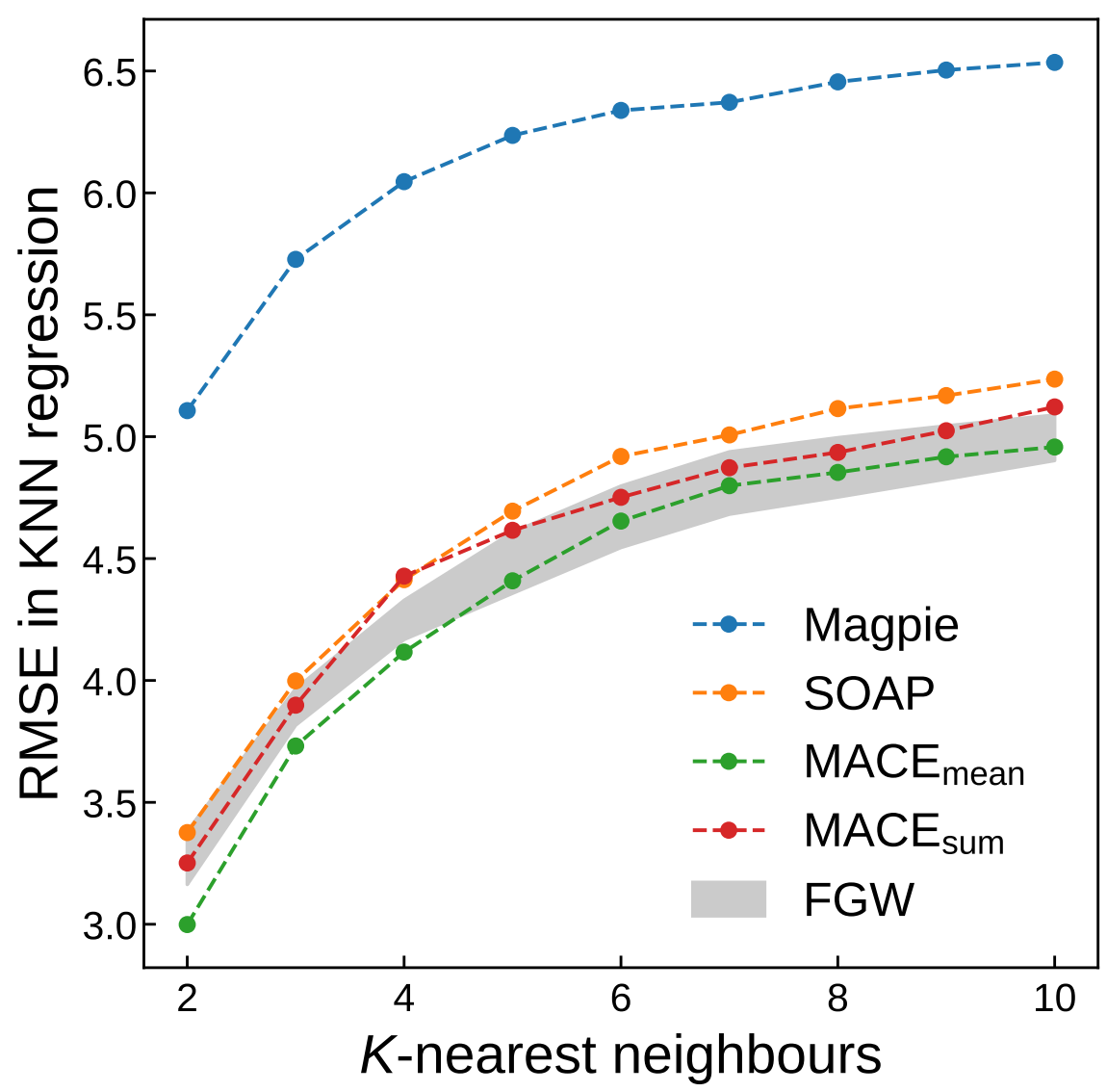

Figure 1 Version of Figure 2b from paper including two pooling approaches for the construction of the MACE graph-level embeddings.

Distributions of enrichment integrals

In Figure 2 we see that the top seed, shown in the main paper, has an unusually large distribution in its enrichment integral - i.e., the different embedding/parameter combinations generally agree on whether the local environment around a high-SLME material is higher or lower than the dataset average, but for this seed there is more disagreement. The low-SLME seed mp-556775 has an expectedly very negative integral for every embedding, while more surprising is mp-23558, which has a high SLME ($30.8\,\%$) yet has consistently negative enrichment integrals. This perhaps suggests that there are few similar materials in the dataset or that those that are are not representative of the full chemical space.

Figure 3 shows the distribution of integrals per embedding. For each we see a very negative outlier: the aforementioned low-SLME seed. CrystaLLM has the highest single integral but the highest average integral is achieved by Oliynyk, hence its use in the materials discovery campaign.

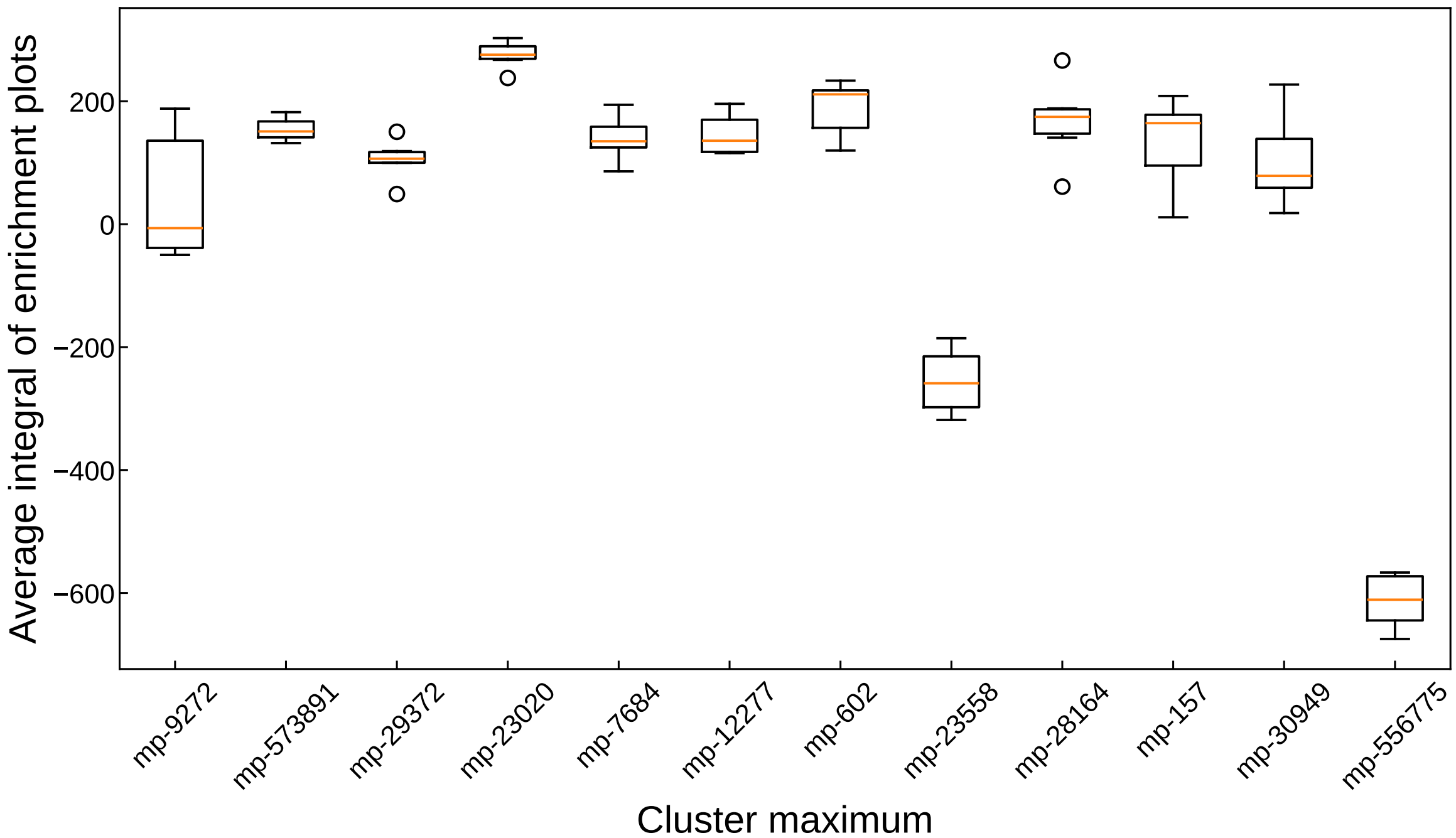

Figure 2 Boxplots for the distribution of 'enrichment integrals' (see main paper for details) for each seed over all feature vectors.

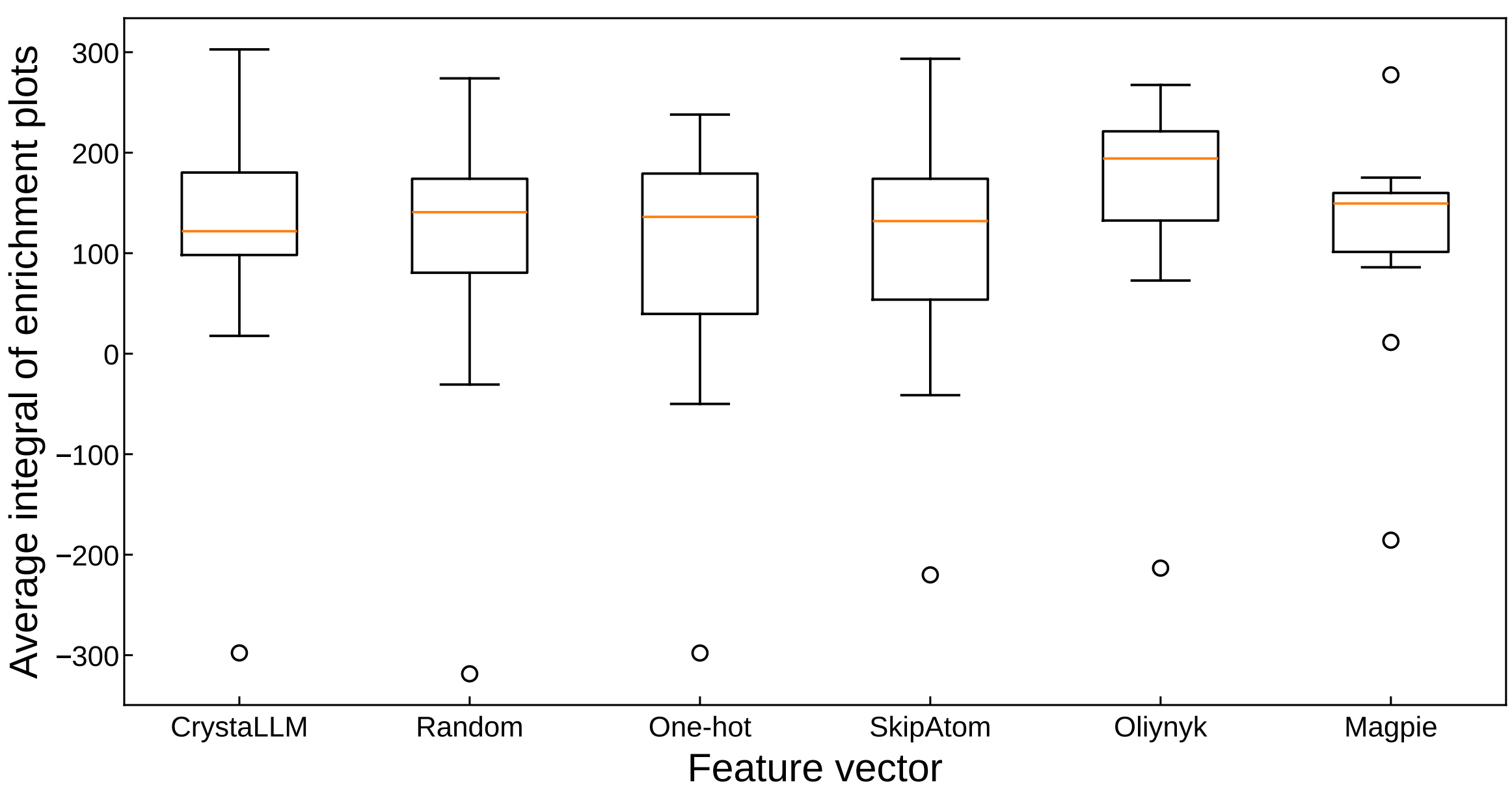

Figure 3 Boxplots for the distribution of 'enrichment integrals' for each feature vector over all seeds.

Exclusion reason	Count
Polymorph of seed	14
Polymorph of candidate	55
Candidate repeated	39
Unstable	281
Wide gap	21
Dimensionality	714
Forbidden elements	43

Table 1 Summary of statistics for each filtering criteria during screening process

Exclusion statistics for screening

1100 materials were considered (101 per seed, then the seeds were removed). These were then filtered to exclude:

- Polymorphs of the seed
- Polymorphs of a given candidate (only the lowest EAH polymorph was kept)
- Repeated candidates (the same candidate suggested by multiple seeds)
- Wide-gap material (band-gap > 2.5 eV)
- Unstable materials (energy above hull > 0.05 eV / atom)
- Low-dimensional (<3) materials (using the approach from Gorai et al.[5] as implemented in `Pymatgen`)
- Materials containing lanthanides, actinides, or radioactive elements

Table 1 shows the numbers of materials by exclusion reasons, though many were excluded for several reasons. A Venn diagram showing the overlap of materials satisfying some of these criteria is given in Figure 4. Clearly the dimensionality was the most limiting criterion, partly due to some of the seeds themselves being 2D, e.g. $MoTe_2$.

References

[1] L. Ward, A. Agrawal, A. Choudhary and C. Wolverton, *npj Computational Materials*, 2016, **2**, 16028.

[2] A. O. Oliynyk, E. Antono, T. D. Sparks, L. Ghadbeigi, M. W. Gaultois, B. Meredig and A. Mar, *Chemistry of Materials*, 2016, **28**, 7324–7331.

[3] L. M. Antunes, R. Grau-Crespo and K. T. Butler, *npj Computational Materials*, 2022, **8**, 44.

[4] L. M. Antunes, K. T. Butler and R. Grau-Crespo, *Nature Communications*, 2024, **15**, 10570.

[5] P. Gorai, E. S. Toberer and V. Stevanović, *Journal of Materials Chemistry A*, 2016, **4**, 11110–11116.

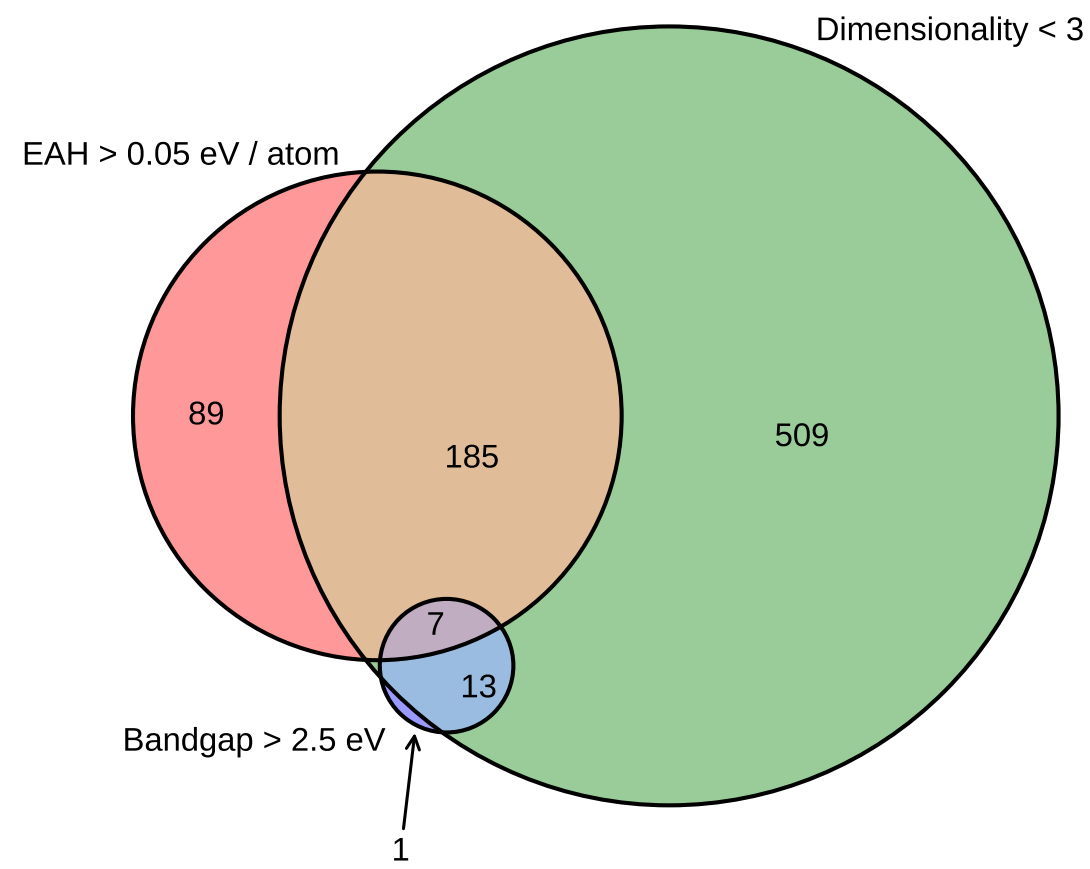

Figure 4 Venn diagram showing the overlap of criteria leading to materials being excluded from the final shortlist
